# Public health round-up

**DOI:** 10.2471/BLT.21.010321

**Published:** 2021-03-01

**Most commonly diagnosed cancer**

A woman receives a breast examination in a mobile mammography unit in the Russian Federation. According to a recent International Agency for Research on Cancer report, breast cancer is now the world’s most commonly diagnosed cancer.

**Figure:**
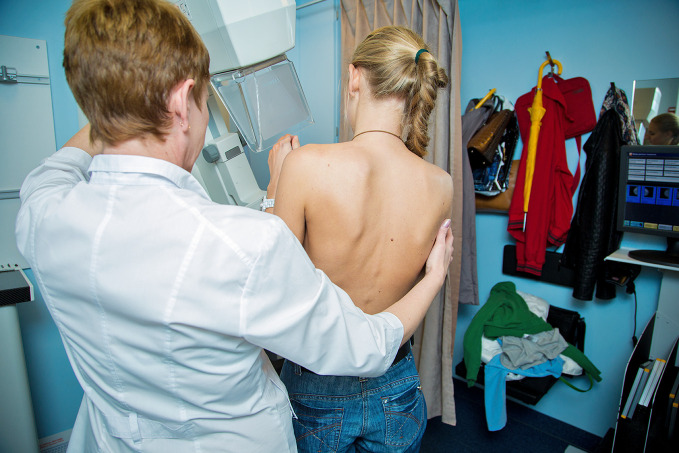
WHO/Sergey Volkov

## Ebola outbreaks

Health authorities in Guinea declared an outbreak of Ebola virus disease on 14 February, after three cases were confirmed by the country’s national laboratory.

Initial investigations found that a nurse working in a health facility in the rural community of Gouéké in N’Zerekore prefecture died on 28 January 2021. Six people who attended her funeral then reported Ebola-like symptoms. As of 14 February, two of those people had died, while the other four had been hospitalized.

In the Democratic Republic of the Congo, the Ministry of Public Health declared an Ebola virus disease outbreak on 7 February after the laboratory confirmation of a case in North Kivu Province. The Butembo branch of the National Institute of Biomedical Research confirmed the presence of the virus in samples taken from a woman who sought treatment at a health centre. The woman subsequently died.

As of 15 February, four people were reported to have been infected with Ebola virus in the Democratic Republic of the Congo, two of whom had died. A total of 149 people had been reported as having been in contact with the woman who first presented with symptoms of the disease. The North Kivu Provincial health authorities have triggered an outbreak response with support from the health ministry and the World Health Organization (WHO).

http://bit.ly/3dc3Kvt

http://bit.ly/3puCFXb

## Yemen nutrition emergency

An estimated 2.3 million children under the age of five are projected to suffer from acute malnutrition in Yemen in 2021. Around 400 000 of those children are expected to suffer from severe acute malnutrition and could die if they do not receive urgent treatment.

This is according to the latest Integrated Food Security Phase Classification Acute Malnutrition report released on 12 February by the Food and Agriculture Organization of the United Nations, the United Nations Children’s Fund (UNICEF), the World Food Programme, WHO and partners.

The agencies called for urgent action, warning that the reported numbers were among the highest levels of severe acute malnutrition recorded in Yemen since the escalation of the conflict in the country in 2015.

http://bit.ly/3bcO2Om

## Globalizing the COVID-19 vaccine roll-out

In a joint statement issued on 10 February, UNICEF and WHO called for global solidarity in the roll-out of coronavirus disease 2019 (COVID-19) vaccines. 

Urging national leaders to look beyond their borders, the agencies pointed out that vaccination strategies based on national interest will give the virus further opportunities to mutate and evade vaccines, cost lives and livelihoods and undermine a global economic recovery.

To ensure that vaccine roll-outs begin in all countries in the first 100 days of 2021, the agencies called on governments that have vaccinated their own health workers and populations at highest risk of severe disease to share vaccines through COVAX, the vaccine pillar of the Access to COVID-19 Tools (ACT) Accelerator, a global collaboration to accelerate the development, production and equitable access to new COVID-19 diagnostics, therapeutics and vaccines.

The agencies urged vaccine manufacturers to allocate the limited vaccine supply equitably and share safety, efficacy and manufacturing data as a priority with WHO for regulatory and policy review. They also called for manufacturers to step up and maximize production and ensure the transfer of technology to other manufacturers who can help scale the global supply.

http://bit.ly/2MYXSLH

## COVAX on track

COVAX announced the signing of an advance purchase agreement for up to 40 million doses of the Pfizer-BioNTech vaccine. Roll-out of the vaccine is to commence with successful execution of supply agreements.

Additionally, COVAX announced that close to 150 million doses of the AstraZeneca/Oxford vaccine are expected to be available in the first quarter of 2021, via existing agreements with the Serum Institute of India and AstraZeneca.

This puts COVAX on track to deliver at least 2 billion doses of needed vaccines by the end of 2021, including at least 1.3 billion doses to 92 lower income economies.

http://bit.ly/3rU8hqv

## COVID-19 vaccine emergency use approval

WHO listed two versions of the AstraZeneca/Oxford COVID-19 vaccine for emergency use, green-lighting the vaccines for global roll-out through the COVAX Facility.

Emergency Use Listing is a prerequisite for COVAX Facility vaccine supply and also allows countries to expedite their own regulatory approval to import and administer COVID-19 vaccines.

http://bit.ly/2OJHrTR

## Breast cancer initiative

WHO hosted the first of a series of consultations on 4 February to establish a new global breast cancer initiative, which it will be launching later in 2021 in collaboration with the International Agency for Research on Cancer (IARC), the International Atomic Energy Agency and other multisectoral partners.

According to statistics released by the IARC in December 2020, breast cancer has overtaken lung cancer as the world’s most commonly diagnosed cancer.

The number of people diagnosed with cancer has nearly doubled in the past two decades, from an estimated 10.0 million in 2000 to 19.3 million in 2020. Today, it is estimated that one in five people worldwide will develop cancer during their lifetime.

http://bit.ly/2MYYctT

## Essential diagnostics

WHO published a new edition of the Essential Diagnostics List, a basket of recommended in vitro diagnostics that should be available at point-of-care and in laboratories in all countries to support timely, life-saving diagnoses.

Published on 29 January, the new edition includes WHO-recommended COVID-19 polymerase chain reaction and antigen tests, expands the suite of tests for vaccine-preventable and infectious diseases and noncommunicable diseases such as cancer and diabetes, and introduces a section on endocrine system diagnostics.

http://bit.ly/3d8U6Ke

## Infodemic research

WHO published a public health research agenda to help tackle the “infodemics” that hamper responses to public health emergencies, as widely observed in the ongoing COVID-19 pandemic.

The five-point agenda covers areas ranging from understanding how information originates, evolves and spreads to improving the effectiveness and timeliness of infodemic countermeasures during acute health events.

The agenda is designed for WHO staff, partners, research agencies and academia to help identify key research and evidence gaps and to support research needed to underpin infodemic management interventions and to facilitate their evaluation.

http://bit.ly/3pfwpSU

**Cover picture**

A midwife listens to the fetal heartbeat of a pregnant woman at a WHO mobile clinic in Garm Abak in the Waras district of Afghanistan.

**Figure:**
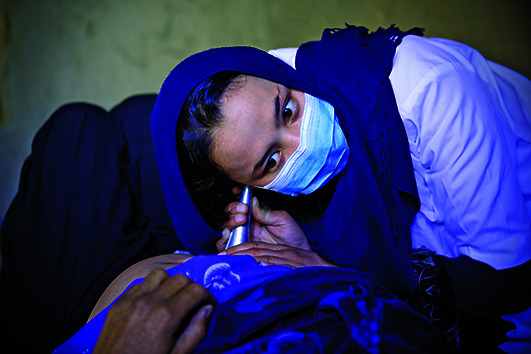
WHO/Rada Akbar

## Keeping score on public health

An estimated four in 10 deaths go unregistered worldwide. This is according to the first global assessment of country health information systems which was released in the SCORE report by WHO in partnership with Bloomberg Philanthropies on 1 February.

The SCORE report notes wide disparities in rates of death registration (in the African Region only one in 10 deaths is officially recorded) and similar disparities in the reporting of cause of death, only two thirds of low-income countries having established a standardized system to report such data.

http://bit.ly/3jT4cjQ

## Building climate resilience

WHO published guidance to help countries build climate-resilient health systems. Released on 10 February, *Quality criteria for health national adaptation plans* provides policy-makers and ministries of health with good practices and quality criteria for health adaptation planning.

The guidance draws on experience gained since 2012 through WHO’s support to countries in developing and implementing such plans, and includes experiences from countries that have started planning or are in the process of implementing developed plans.

http://bit.ly/2NwyVH1

## New paediatric cancer tools

WHO released a suite of tools designed to help countries improve diagnosis and treatment of paediatric cancer. Released on 15 February, the tools include a guide for policy-makers, cancer control programme managers and hospital managers; an assessment tool to inform implementation; and a multilingual online portal for information-sharing. The new tools will support countries with implementation of the CureAll approach, adopted by WHO’s Global Initiative for Childhood Cancer which aims to achieve at least 60% survival for childhood cancer globally by 2030.

http://bit.ly/3da8lOW

## Investigating COVID-19 origins

The independent expert team to study the origins of the COVID-19 virus completed its trip to China in the second week of February. As of 12 February, the team was working on a summary report and planned to publish a full report in the following weeks.

http://bit.ly/2Nt2U2R

**Looking ahead**

15–26 March. 65th session of the Commission on the Status of Women. http://bit.ly/37fULpo

22–24 March. 16th coordination meeting of the WHO Radiation Emergency Medical Preparedness and Assistance network. http://bit.ly/3nIQArw

23–25 March. Meeting of the Strategic Advisory Group of Experts on Immunization.

http://bit.ly/3puFCqJ

